# Seneca Valley virus 3C^pro^ degrades heterogeneous nuclear ribonucleoprotein A1 to facilitate viral replication

**DOI:** 10.1080/21505594.2021.2014681

**Published:** 2021-12-18

**Authors:** Jiangwei Song, Dan Wang, Rong Quan, Jue Liu

**Affiliations:** a Beijing Key Laboratory for Prevention and Control of Infectious Diseases in Livestock and Poultry, Institute of Animal Husbandry and Veterinary Medicine, Beijing Academy of Agriculture and Forestry Sciences, Beijing, China; b College of Veterinary Medicine, Yangzhou University, Yangzhou, China; c Jiangsu Co-innovation Center for Prevention and Control of Important Animal Infectious Diseases and Zoonoses, Yangzhou University, Yangzhou, China

**Keywords:** Seneca valley virus (SVV), hnRNP A1, degradation, 3C protease, replication

## Abstract

Seneca Valley virus (SVV) is a recently-identified important pathogen that is closely related to idiopathic vesicular disease in swine. Infection of SVV has been shown to induce a variety of cellular factors and their activations are essential for viral replication, but whether heterogeneous nuclear ribonucleoprotein A1 (hnRNP A1) involved in SVV replication is unknown. The cytoplasmic redistribution of hnRNP A1 is considered to play an important role in the virus life cycle. Here, we demonstrated that SVV infection can promote redistribution of the nucleocytoplasmic shuttling RNA-binding protein hnRNP A1 to the cytoplasm from the nucleus, whereas hnRNP A1 remained mainly in the nucleus of mock-infected cells. siRNA-mediated knockdown of the gene encoding hnRNP A1 attenuated viral replication as evidenced by decreased viral protein expression and virus production, whereas its overexpression enhanced replication. Moreover, infection with SVV induced the degradation of hnRNP A1, and viral 3 C protease (3 C^pro^) was found to be responsible for its degradation and translocation. Further studies demonstrated that 3 C^pro^ induced hnRNP A1 degradation through its protease activity, via the proteasome pathway. This degradation could be attenuated by a proteasome inhibitor (MG132) and inactivation of the conserved catalytic box in 3 C^pro^. Taken together, these results presented here reveal that SVV 3 C protease targets cellular hnRNP A1 for its degradation and translocation, which is utilized by SVV to aid viral replication, thereby highlighting the control potential of strategies for infection of SVV.

## Introduction

Seneca Valley virus (SVV) was detected originally as a contaminant of PER.C6 cell cultures in the United States in 2002 [1,2]. Subsequently, the etiological agent of idiopathic vesicular disease in pigs was confirmed as SVV infections in Canada in 2007 [3]. To date, a wealth of cases of SVV infection have been reported in many pig-rearing countries, such as Brazil [4], USA [5], Thailand [6], Colombia [7], China [8]. Infection with SVV can cause idiopathic vesicular disease-like clinicopathological signs, such as vesicular lesiones on the oral, coronary bands, snout, and hooves, and has increasingly threatened to global pig industry [1]. In addition, SVV is a promising candidate for neuroendocrine cancer therapy due to its oncolytic activity [4].

SVV, belonging to the genus *Senecavirus* of the family *Picornaviridae* [2,5], is a non-enveloped, positive-sense, single-stranded RNA virus. The SVV genome is approximately 7,200 bases and encodes a polyprotein that is cleaved into four structural proteins and eight nonstructural proteins by viral proteases 2A, 3 C (3 C^pro^), and cellular proteases [2]. Among them, 3 C^pro^ is a multifunctional protein that it can degrade and cleave numerous cellular proteins directly, and which has been identified as a deubiquitinase that inhibits type I interferon production [6–8]. SVV 3 C^pro^ is responsible for the degradation of retinoic acid-inducible gene I (RIG-I) which inhibits type I interferon production [8]. SVV 3 C^pro^ disrupts the eIF4GI-G3BP1 interaction, thus inhibits stress granule formation [9]. SVV 3 C^pro^ cleaves poly(A)-binding protein cytoplasmic 1 (PABPC1), which is dependent on the protease activity at sites 48 and 160, and facilitates viral replication [10]. Moreover, protease activity is essential for the induction of apoptosis and cleavage of nuclear factor kappa B (NF-κB) p65 and poly (ADP-ribose) polymerase (PARP) [11]. SVV 3 C^pro^ can induce apoptosis by activating caspase-8, caspase-9, and caspase-3 [12].

The heterogeneous nuclear ribonucleoproteins (hnRNPs) family are RNA-binding proteins [13]. More than 20 hnRNPs have been identified, named hnRNP A to hnRNP U, the majority of which are associated with precursor messenger RNA (pre-mRNA) splicing and mRNA metabolism [13,14]. Among these hnRNPs, hnRNP A1, which is involved in pre-mRNA splicing and transportation of RNA [15,16], is the best-known member of this protein family and includes two RNA-binding domains (RBDs) and a C-terminal glycine-rich domain that is essential for protein-protein interaction [17–19]. Cellular hnRNP A1 binds to numerous viral proteins and regulates virus replication, including the nucleocapsid protein of the SARS coronavirus and porcine epidemic diarrhea virus (PEDV) [20,21]. hnRNP A1 binds to the avian reovirus (ARV) p17 protein and modulates nucleocytoplasmic shuttling of p17 [22]. Furthermore, hnRNP A1 also interacts with the viral RNA element, which directly binds to an RNA sequence in the human papillomavirus 16 (HPV16) E7 coding region [23].

The replication of picornavirus mainly takes place in the cytoplasm of the host cells, and utilizes cellular machinery to facilitate viral RNA transcription, splicing, and translation [24–26]. hnRNP A1 in the nucleus shuttles to the cytoplasm which is a regulated process in virus-infected cells and cellular stress [21,22,25]. The cytoplasmic redistribution of hnRNP A1 plays critical roles in viral life cycles, such as the formation of the viral ribonucleoprotein complex and IRES-mediated translation initiation [24,26–29]. The RBDs and protein-binding region of hnRNP A1 can mediate formation of the mouse hepatitis virus (MHV) ribonucleoprotein complex that is involved in viral RNA transcription [28,29]. Moreover, hnRNP A1 binds to the viral 5ʹ untranslated region (UTR), which regulates IRES-dependent translation to facilitate viral replication [24–26].

Infection of SVV has been shown to induce a variety of cellular factors and their activations are essential for viral replication, but whether hnRNP A1 is involved in SVV replication through regulating IRES-dependent translation as observed for some other picornaviruses [24–26] is still unclear. Here, we found that infection with SVV induced the redistribution of hnRNPA1 to the cytoplasm. Furthernore, SVV-mediated degradation of hnRNPA1 was modulated by the protease activity of the 3 C protease through the proteasome pathway. Inhibition of hnRNPA1 expression greatly decreased SVV replication, whereas overexpression of hnRNP A1 enhanced SVV replication. These results indicated that cellular hnRNP A1 contributes to SSV replication via its degradation and translocation mediated by protease activity of SVV 3 C protein.

## Materials and methods

### Cells, viruses, antibodies, and chemical reagents

BHK-21 cells and PK-15 cells were cultured in Dulbecco’s modified Eagle’s medium (DMEM) (Invitrogen, CA, USA) containing 10% fetal bovine serum (FBS) (Invitrogen) at 37°C in a humidified 5% CO_2_ incubator. The SVV strain CHhb17 [30] was used in this study. Mouse monoclonal antibody raised against VP1 proten was from our laboratory. Mouse anti-β-actin antibody (A1978) and mouse anti-dsRNA antibody (MABE1134) were obtained from Sigma-Aldrich (St. Louis, MO, USA). Rabbit anti-hnRNP A1 (ab177152), rabbit anti-histone H3 antibody (ab183902), and mouse anti-GFP (ab127417) were obtained from Abcam (Cambridge, MA, USA). Horseradish peroxidase (HRP)-conjugated goat anti-rabbit (ab205719) and anti-mouse (205,718) secondary antibodies were obtained from Abcam. Alexa-568-conjugated goat anti-mouse and -rabbit secondary antibodies were obtained from Molecular Probes (Invitrogen). MG132 and Z-VAD-FMK were obtained from Selleck Chemicals (Shanghai, China).

### Plasmid construction

The hamster hnRNP A1 gene was cloned from BHK-21 cells and recombined into the lentivirus vector pWPXL (Addgen, 12,257) and pCMV-HA (Clontech, 631,604). A series of GFP-infused SVV proteins were constructed into the vector pEGFP-C1 (Clontech, U55763) by cloning the gene fragment from SVV-infected BHK-21 cells. Mutagenesis PCR was performed on GFP-3 C using KOD DNA polymerase (TOYOBO, KFX-201) to generate GFP-3 C-H48A, GFP-3 C-C160A, GFP-3 C-DM (H48A and C160A double mutants). All the constructs were confirmed by sequencing. The primers used for this study are displayed in Table 1.

**Table 1.** Primers used in this study

Primers^a^		Sequence (5ʹ-3ʹ)^b^	Restriction site
GFP-Lpro-F GFP-Lpro-R GFP-VP4-F GFP-VP4-R GFP-VP2-F GFP-VP2-R GFP-VP3-F GFP-VP3-R GFP-VP1-F GFP-VP1-R GFP-2B-F GFP-2B-R GFP-2 C-F GFP-2 C-R GFP-3A-F GFP-3A-R GFP-3C-F GFP-3C-R GFP-3D-F GFP-3D-R hnRNP A1-GFP-F hnRNP A1-GFP-R HA-hnRNP A1-F HA-hnRNP A1-R	TCGAGCTCAAGCTTCGAATTCTATGCAGAACTCTCATTTTTCTTT TTATCTAGATCCGGTGGATCCTTACTGCAGCTCGTATACGATGTCC TCGAGCTCAAGCTTCGAATTCTGGTAATGTTCAGACAACCTCA TTATCTAGATCCGGTGGATCCTTATTTGAGGTAGCCAAGAGGGTT TCGAGCTCAAGCTTCGAATTCTGATCACAATACCGAAGAAATG TTATCTAGATCCGGTGGATCCTTACTGTTCCTCGTCCGTCCCGGT TCGAGCTCAAGCTTCGAATTCTGGGCCCATTCCCACAGCACCCAGAGAAA TTATCTAGATCCGGTGGATCCTTAGTGGAACACGTAGGAAGGATTA TCGAGCTCAAGCTTCGAATTCTTCCACCGACAACGCCGAGACTGGTGTTA TTATCTAGATCCGGTGGATCCTTATTGCATCAGCATCTTTTGCTT TCGAGCTCAAGCTTCGAATTCTGGCCCTGCTTCTGACAACCCA TTATCTAGATCCGGTGGATCCTTATTGCATCTTGAACAGCTTTCG TCGAGCTCAAGCTTCGAATTCTGGACCCATGGATACAGTCAAAG TTATCTAGATCCGGTGGATCCTTACTGTAGAACCAGAGTCTGCATATT TCGAGCTCAAGCTTCGAATTCTAGCCCTAACGAGAACGACGACAC TTATCTAGATCCGGTGGATCCTTACTCGCTCCTAGGCGCTTTAGCA TCGAGCTCAAGCTTCGAATTCTCAGCCCAACGTGGACATGGGCT TATCTAGATCCGGTGGATCCTTATTGCATTGTAGCCAGAGGCTCA TCGAGCTCAAGCTTCGAATTCTGGACTAATGACTGAGCTAGAGC TTATCTAGATCCGGTGGATCCTTAGTCGAACAAGGCCCTCCATCTT GAGGTTTAAACTACGGGATCCAATGTCTAAGTCCGAGTCACCCA ACCGGTAGCGCTAGGACGCGTAAGAACCTCCTGCCACTGCCATAGCT TGGCCATGGAGGCCCGAATTCGGATGTCTAAGTCCGAGTCACCCA GATCCCCGCGGCCGCGGTACC TTAGAACCTCCTGCCACTGC		EcoRI BamHI EcoRI BamHI EcoRI BamHI EcoRI BamHI EcoRI BamHI EcoRI BamHI EcoRI BamHI EcoRI BamHI EcoRI BamHI EcoRI BamHI BamHI MluI EcoRI KpnI

a F denotes forward PCR primer; R denotes reverse PCR primer. b Restriction sites are underlined.

### Western blotting

BHK-21 cells and PK-15 cells were lysed using lysis buffer (0.5% NP-40, 50 mM Tris, 0.5 mM EDTA, 150 mM NaCl) with protease inhibitor (1 mM phenylmethylsulfonyl fluoride, PMSF) on ice for 30 min, samples were incubated at 4°C with rotation for 30 min. The lysates were clarified by centrifugation at 12,000 rpm for 30 min. Protein samples were quantified using a bicinchoninic acid kit (Thermo Fisher, 23,225). Equal amounts of proteins were separated using sodium dodecyl sulfate polyacrylamide gel electrophoresis (SDS-PAGE) and then transferred onto nitrocellulose (NC) membranes (PALL, FL, USA). Membranes were blocked with TBST buffer containing 5% nonfat milk. The membranes were incubated with specific primary antibodies and then incubated with HRP-conjugated secondardy antibodies. Bound proteins were visualized using chemiluminescence detection reagents (Thermo Fisher, 34,096).

### Quantitative reverse transcription-polymerase chain reaction (qRT-PCR)

Total RNA was extracted from infected BHK-21 and PK-15 cells using TRIzol reagent (Invitrogen). qRT-PCR was conducted using SYBR qPCR Master Mix (Vazyme, China). β-Actin served as the reference gene. Data were expressed as relative fold change using the comparative cycle threshold (CT) (2^−ΔΔCT^) method [31]. The primers used for the qRT-PCR were displayed in Table 1.

### Immunofluorescence assay (IFA)

Immunofluorescence assays were performed as previously described [32]. Briefly, BHK-21 cells grown on coverslips were fixed with 4% paraformaldehyde, permeabilized with 0.1% Triton X-100, and then blocked with 2% BSA-PBS. Primary and secondary antibodies were sequentially incubated followed by incubation with 4ʹ,6-diamidino-2-phenylindole (DAPI). The cells were examined under a Nikon A1 confocal microscopy.

### Nuclear and cytoplasmic fractionation

Mock- or SVV-infected BHK-21 cells were harvested and nuclear and cytoplasmic fractions were fractionated using the nuclear and cytoplasmic extraction reagents (Thermo Fisher, 78,833) in accordance with the manufacturer’s instructions. Histone-H3 was used as a marker for nuclear internal control, and β-actin was used as a marker for cytoplasmic internal control in Western blot analysis.

### Lentivirus packaging

HEK293FT cells were co-transfected with 1.18 µg pWPXL (#12,257), 0.47 µg pMD2.G (#12,259), and 2.35 µg psPAX2 (#12,260) to rescue the lentivirus. The supernatants were harvested and concentrated using an Amicon Ultra-100 centrifugal filter unit (Millipore, USA). BHK-21 cells were transduced with a lentivirus containing 1 µg/ml polybrene.

### Virus infection and TCID_50_ assay

BHK-21 cells and PK-15 cells were infected with SVV strain CHhb17 and incubated with DMEM containing 2% FBS. The cell supernatants were harvested at the indicated times after SVV infection. The virus titers were assayed by limiting dilutions using the Spearman and Karber’s method and represented as the tissue culture infectious dose 50 (TCID_50_).

### Knockdown of hnRNP A1 through RNA interference

The siRNAs targeting hnRNP A1 were synthesized by GenePharma (Suzhou, China): si-1 (sense, 5ʹ-CCCUGUCAAAGCAAGAGAUTT-3ʹ; antisense, 5ʹ-AUCUCUUGCUUUGACAGGGTT-3ʹ), si-2 (sense, 5ʹ-GGACCAAUGAAGGGUGGAATT-3ʹ; antisense, 5ʹ-UUCCACCCUUCAUUGGUCCTT-3ʹ). The knockdown of hnRNP A1 in BHK-21 cells was conducted by the transfection of hnRNP A1 siRNA using Lipofectamine RNAiMAX (Thermo Fisher, 13,778,150). The siRNA (sense, 5ʹ-UUCUCCGAACGUGUCACGUTT-3ʹ; antisense, 5ʹ-ACGUGACACGUUCGGAGAATT-3ʹ) served as a negative control.

### Statistical analysis

Statistical significance used in this work was evaluated using GraphPad Prism (version 5.0; La Jolla, CA, USA). All data are reported as mean ± standard deviation (SD), with a *P* < 0.05 being set statistical significance.

## Results

### SVV infection induces hnRNP A1 degradation

**Figure 1.**
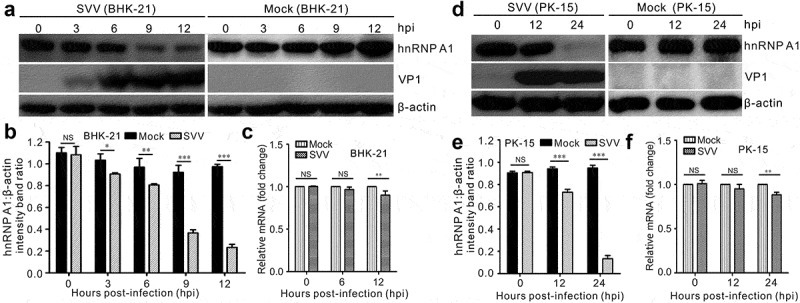
SVV infection induces hnRNP A1 degradation. (a, d) BHK-21cells and PK-15 cells after SVV infection were collected at 0, 3, 6, 9, and 12 hpi, and 0, 12, and 24 hpi, respectively. The levels of cellular hnRNP A1 were analyzed by Western blotting. (b, e) The expression of cellular hnRNP A1 normalized against β-actin. (c, f) BHK-21 and PK-15 cells were infected with SVV, respectively. Infected cells were harvested, and the transcriptional levels of hnRNP A1 were calculated by qRT-PCR. Expression of hnRNP A1 was normalized to the actin mRNA level. GraphPad Prism was used for statistical analysis. The data are represented as the mean ± SD from three independent experiments (*P < 0.05; **P < 0.01; ***P < 0.001; NS, not significant)

Considering the importance of hnRNP A1 in virus replication [22–24,26–28,33], we analyzed the expression of hnRNP A1 in both BHK-21 and PK-15 cells after SVV infection. Western blotting and RT-qPCR were ulitized to analyze the expression level of hnRNP A1 over the course of SVV infection. The results showed that hnRNP A1 protein levels were dramatically reduced during SVV infection (Figure 1a, 1d). This was observed, in particular, in SVV-infected cultured BHK-21 cells at 9 h post-infection (Figure 1a-1b) and SVV-infected PK-15 cells at 12 h post-infection (Figure 1d-1e). The mRNA transcription level of hnRNP A1 was also decreased significantly (Figure 1c, 1f), which agreed with the results of the Western blotting (Figure 1a, 1d). These results suggest that infection with SVV induced hnRNP A1 degradation in the cultured cells.

### SVV infection induces translocation of hnRNP A1 to the cytoplasm from the nucleus

**Figure 2.**
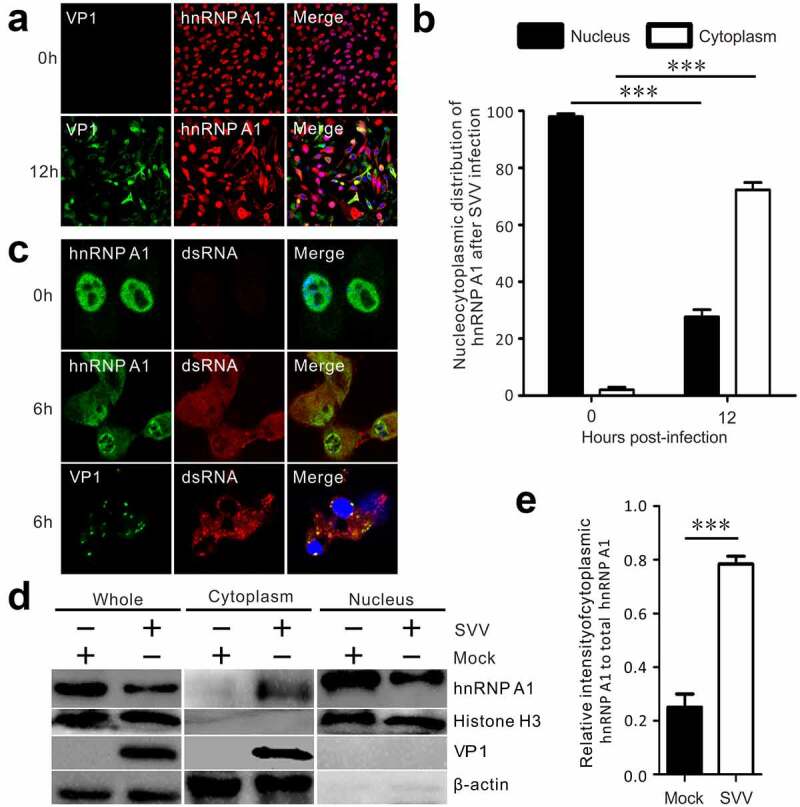
SVV infection induces translocation of hnRNP A1 to the cytoplasm from the nucleus. (a) At 12 h after SVV infection, the cultured BHK-21 cells were stained with hnRNP A1 antibody (red), SVV VP1 antibody (green), and DAPI (blue), then examined by confocal microscopy. (b) Statistical analysis for the percentage redistribution of hnRNP A1 after SVV infection. GraphPad Prism was used for statistical analysis, and the data was expressed as mean ± SD from three independent experiments. (***P < 0.001). (c) At 6 h after SVV infection, the cultured BHK-21 cells were stained with the hnRNP A1 antibody (green), SVV VP1 antibody (green), dsRNA antibody (red), and DAPI (blue), then examined by confocal microscopy. (d) Cell cytoplasmic and nuclear components were extracted from SVV-infected cells. Samples were detected by Western blotting with antibodies against hnRNP A1, VP1, histone H3, and β-actin. (e) The relative gray intensity of cytoplasmic hnRNP A1 was normalized against total hnRNP A1 and quantified by ImageJ; GraphPad Prism was used for statistical analysis, and the data was expressed as mean ±SD from three independent experiments. (***P < 0.001)

To further decipher the relationship between hnRNP A1 and SVV replication, immunofluorescence assays were performed. The results showed that hnRNP A1 was primarily localized in the nucleus of mock-infected cells (Figure 2a), and most hnRNP A1 was translocated to the cytoplasm from the nucleus of SVV-infected BHK-21 cells (Figure 2a). The nucleocytoplasmic redistribution of hnRNP A1 was statistically increased after SVV infection (Figure 2b). We also used antibodies against viral double-stranded RNA (dsRNA) to observe the colocalization of cellular hnRNP A1 and viral dsRNA. After SVV infection, hnRNP A1 relocalized to the cytoplasm and was colocalized with viral dsRNA (Figure 2c). Moreover, VP1 partially colocalized with viral dsRNA (Figure 2c). In addition, cytoplasmic and nuclear fractions were extracted to further evaluate redistribution after SVV infection (Figure 2d). Histone subunits H3 and β-actin served as internal references for nuclear and cytoplasmic fractionation, respectively. The distribution of cytoplasmic hnRNP A1 was significantly enhanced in the SVV-infected BHK-21 cells compared to that in mock-infected cells (Figure 2d-2e). These findings suggest that SVV infection promoted the relocalization of hnRNP A1 to the cytoplasm from the nuclear.

### 3 C^pro^ induces hnRNP A1 degradation and redistribution

**Figure 3.**
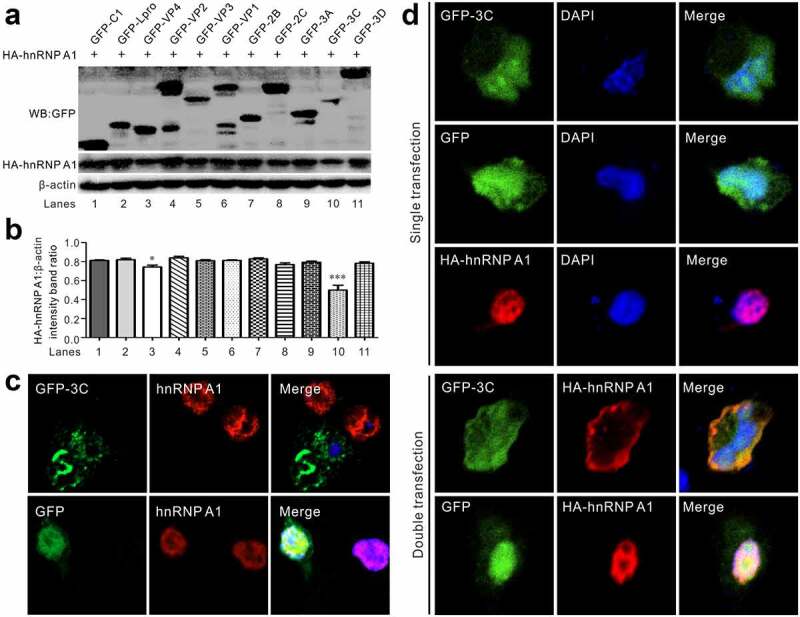
SVV 3C was responsible for hnRNP A1 degradation and translocation. (a) Western blot analysis of BHK-21 cells cotransfected with GFP-tagged SVV protein-expressing plasmids and HA-hnRNP A1. (b) Rations to β-actin of three independent experiments (*P < 0.05; ***P < 0.001). Image J was employed to quantify the protein levels. (c) Fluorescence for transfection of plasmids expressing GFP (green) and GFP-3C(green), endogenous hnRNP A1 (red) was stained with the hnRNP A1 antibody, and DAPI (blue) in BHK-21 cells, and then examined by confocal microscopy. (d) Fluorescence to analyze transfection of plasmids expressing GFP (green), GFP-3C (green), and HA hnRNP A1 were stained with the HA-tagged antibody (red), and DAPI (blue) in BHK-21 cells, and then examined by confocal microscope

To gain insight into the mechanism of SVV-infection-induced hnRNP A1 degradation and redistribution, we initially screened for the viral protein responsible for hnRNP A1 degradation by Western blot and immunofluorescence observation (Figure 3a-3c). A series of viral protein-expressing plasmids were constructed and fused to a GFP tag. After co-transfection of GFP-tagged SVV protein-expressing plasmids and HA-hnRNP A1, Western blotting showed that the levels of hnRNP A1 in GFP-3 C-transfected BHK-21 cells significantly decreased compared to cells transfected with the GFP vector or other viral protein-expressing plasmids (Figure 3a-3b). In addition, GFP-tagged Lpro also degraded hnRNP A1. We then investigated endogenous degradation using an antibody against hnRNP A1 in an immunofluorescence assay. The immunofluorescence signals of endogenous hnRNP A1 (red) in GFP-3 C-transfected BHK-21 cells disappeared, while immunofluorescence (red) in GFP vector-transfected cells was not influenced (Figure 3c). This indicated that 3 C^pro^ could induce hnRNP A1 degradation *in vivo*. Finally, we co-expressed 3 C^pro^ and hnRNP A1 for in vitro confocal microscopy. Based on the results of SVV-infected BHK-21 cells (Figure 3a), immunofluorescence showed that GFP-3 C induced translocation of HA-hnRNP A1 to the cytoplasm from the nucleus (Figure 3d). When expressed alone, HA-hnRNP A1 was mainly localized to the nucleus, whereas upon co-expression with GFP-3 C, HA-hnRNP A1 was absent from the nucleus and relocalized to the cytoplasm (Figure 3d). In contrast, GFP did not respond to HA-hnRNP A1 (Figure 3d). Together, these results suggest that 3 C^pro^ can induce hnRNP A1 degradation and redistribution.

### SVV 3 C^pro^ induces hnRNP A1 degradation via the proteasome pathway

**Figure 4.**
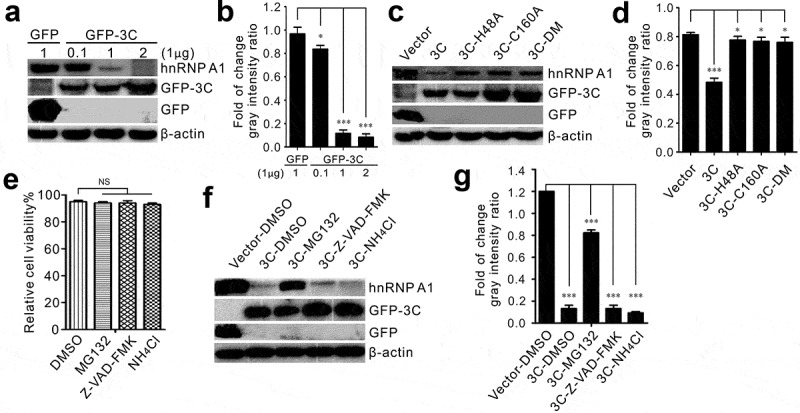
Enzyme activity of 3C was essential for hnRNP A1 degradation. (a) BHK-21 cells were cotransfected with HA-hnRNP A (1 μg) and an increasing amount of GFP-3C (0.1, 1, 2 μg) or empty vector (1 μg), respectively. The cell lysates were analyzed at 24 h post-transfection using Western blot. (b) The ratios to β-actin of three independent experiments of (A) (*P < 0.05; ***P < 0.001). Image J was employed to quantify the protein levels. (c) BHK-21 cells were transfected for 24 h with GFP-3C-WT, GFP-3C-H48A, GFP-3C-C160A, GFP-3C-DM, or empty vector, respectively. The cell lysates were analyzed at 24 h post-transfection using Western blot. (d) The ratios to β-actin of three independent experiments of (C) (*P < 0.05; ***P < 0.001). Image J was employed to quantify the protein levels. (e) The viability of BHK-21 cells after treatment with chemical reagents was tested by CCK-8 assay (NS, not significant). (f) BHK-21 cells were cotransfected with HA-hnRNP A1 and GFP-3C or empty vector, respectively. Cells were treated with DMSO, Z-VAD-FMK (50 μM), MG132 (10 μM), and NH_4_Cl (10 mM). The cell lysates were analyzed at 24 h post-transfection using Western blot. (g) The ratios to β-actin of three independent experiments of (F) (***P < 0.001). Image J was employed to quantify the protein levels

The 3 C^pro^ protease activity involves a variety of cellular protein degradation and cleavage [6,8,10,34]. The conserved catalytic residues of 3 C^pro^ contain histidine (^H^3C^48^) and cysteine (^C^3C^160^) [2]. Therefore, we investigated whether protease activity was required for 3 C^pro^-induced degradation of hnRNP A1. First, BHK-21 cells were cotransfected using different doses, with HA-hnRNP A1 and GFP-3 C plasmids, or an empty vector. Degradation was analyzed by Western blotting at 24 h after transfection. The expression level of hnRNP A1 was found to be greatly reduced in a dose-dependent fashion in the presence of GFP-3 C (Figure 4a-4b). Next, we examined the effect of protease activity on hnRNP A1 degradation. The investigation focused on the three mutants, namely, GFP-3 C-H48A, GFP-3 C-C160A, and GFP-3 C-DM (H48A-C160A). Cells were cotransfected with HA-hnRNP A1 with GFP-3 C, GFP-3 C, GFP-3 C-H48A, GFP-3 C-C160A, and GFP-3 C-DM plasmids. As expected, compared to transfection of the wild-type GFP-3 C, degradation was inhibited after co-transfection with the three mutant types of GFP-3 C (Figure 4c-4d). Thus, our findings provide strong evidence that the catalytic histidine (^H^3C^48^) and cysteine (^C^3C^160^) residues of 3 C^pro^ were responsible for the degradation, and that the loss of its protease activity abolished the degradation (Figure 4c-4d). Finally, MG132, Z-VAD-FMK, and NH_4_Cl were used to evaluate the pathway involved in 3 C^pro^-mediated degradation of hnRNP A1. The viability of the cells was not influenced by treatment with the chemical reagents (Figure 4e). The degradation was dramatically inhibited after treatment with the proteasome inhibitor MG132 (figure 4f-4g). The protein abundance of hnRNP A1 was not influenced after treatment with the caspase inhibitor Z-VAD-FMK or the lysosome inhibitor NH_4_Cl (Figure 4f-4g). These results indicate that 3 C^pro^ induces degradation of hnRNP A1 through the proteasome pathway.

### Knockdown of hnRNP A1 inhibits SVV replication

**Figure 5.**
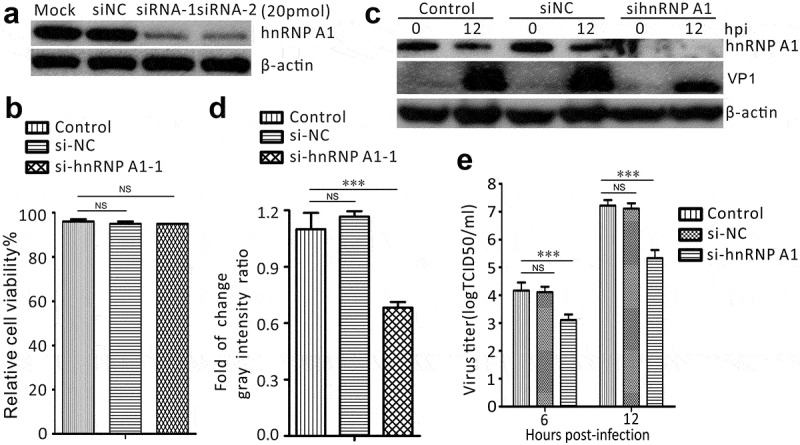
Downregulation of the expression of hnRNP A1 inhibits SVV replication in BHK-21 cells. (a) The silencing efficiency of hnRNP A1 in BHK-21 cells was measured by Western blot at 48 h after siRNA transfection at a concentration of 20 pM, and with no siRNA transfection. (b) The viability of siRNA-transfected BHK-21 cells and normal BHK-21 cells, tested using the CCK-8 assay (NS, not significant). The viability was analyzed using GraphPad Prism, and data were represented as the mean ± SD of three independent experiments (NS, no significant). (c) Western blotting analyzes the levels of VP1 protein and hnRNP A1 in siRNA-transfected BHK-21 cells after SVV infection. (d) The relative gray intensity for VP1 normalized against β-actin was quantified using ImageJ. The results are reported as the mean ± SD of three independent experiments (***P < 0.001; NS, not significant). (e) siRNA transfected and non-siRNA transfected BHK-21 cells were infected with SVV; viral titers in the supernatants at 6 and 12 h post-infection were determined by TCID_50_ assay. The virus titer was examined using GraphPad Prism, and data were reported as mean ± SD (***P < 0.001; NS, not significant)

Given that SVV infection induces hnRNP A1 redistribution, we downregulated the expression of hnRNP A1 using small interfering RNAs (siRNAs) to evaluate its effect on the replication of SVV. BHK-21 cells were transfected with two siRNAs targeting the differential coding regions of hnRNP A1, and Western blotting showed an efficient knockdown of 20 pmol in comparison with mock-transfected cells and negative control siRNA (siNC)-transfected cells (Figure 5a). Importantly, siRNA-treatment did not affect the viability of the transfected cells (Figure 5b). The expression of SVV VP1 protein was reduced in hnRNP A1-knockdown cells, compared to that in siNC-transfected cells and mock-transfected cells, as determined by Western blotting (Figure 5c-5d). Consistent with this, hnRNP A1 knockdown resulted in a significant reduction in the virus titer at 6 and 12 h after SVV infection (hpi) (Figure 5e). These results indicated that hnRNP A1 is an essential host factor involved in SVV replication.

### Overexpression of hnRNP A1 enhances SVV replication

**Figure 6.**
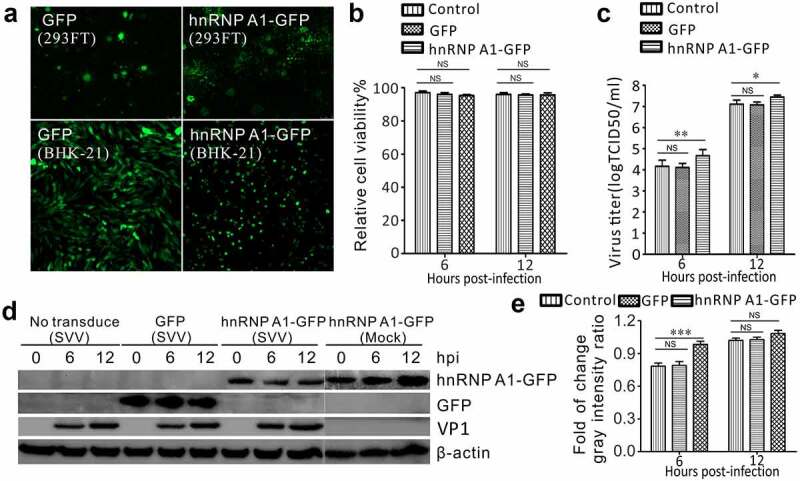
Ectopic expression of hnRNP A1 enhanced SVV replication in BHK-21 cells. (a) Fluorescence of rescued lentiviruses in HEK-293 FT transfected cells and lentivirus-transduced BHK-21 cells expressing hnRNP A1-GFP and GFP. (b) Cell viability was examined by CCK-8 assay in lentiviruses-transduced BHK-21 cells (NS, not significant). (c) Viral titers (tested by TCID_50_ assay) in the supernatants of SVV infected cells after 6 and 12 hpi (*P < 0.05; **P < 0.01; NS, not significant). (d) Western blot analysis of VP1, hnRNP A1-GFP, GFP, and β-actin in lysates collected from SVV-infected cells at 0, 6, and 12 h post-infection, and using anti-VP1 and anti-GFP antibodies. (e) The relative gray intensity for VP1 normalized against β-actin was quantified using ImageJ. The results are reported as the mean ± SD from three independent experiments. (***P < 0.001; NS, not significant)

To confirm the effect of hnRNP A1 overexpression on SVV infection, we established BHK-21 cells that stably overexpressed hnRNP A1-GFP and GFP cell lines using a lentivirus delivery system (Figure 6a). Lentiviral plasmids expressing hnRNP A1-GFP and GFP were successfully rescued in co-transfected HEK293FT cells (Figure 6a). Importantly, BHK-21 cells stably overexpressing hnRNP A1-GFP and GFP were successfully transduced with lentivirus (Figure 6a). The viability of lentivirus-transduced cells and non-transduced cells showed no difference (Figure 6b). The supernatants of SVV-infected lentivirus-transduced cells were collected at 6 and 12 hpi, and viral titers were tested using TCID_50_ assays. Compared with GFP-transduced cells and non-transduced cells, hnRNP A1-GFP transduced cells showed an elevated virus titer at 6 and 12 hpi (Figure 6c). In association with increased virus titer, the levels of VP1 protein of SVV were enhanced in hnRNP A1-overexpressed cells (Figure 6d-6e). Taken together, these results confirm that hnRNP A1 overexpression facilitates SVV replication.

## Discussion

The hnRNP family is predominantly located in the nucleus. After stimulation, they relocalize to the cytoplasm, and this process is regarded as being responsible for stress stimuli such as virus infection [22,35], nucleolar stresses [36], and mitochondrial retrograde response [37]. The hnRNP family, such as hnRNP A1, hnRNP C, hnRNP K, and hnRNP M, are pivotal cellular factors for picornavirus replication [24,38–40]. Cytoplasmic relocalization of hnRNP A1 controls viral RNA synthesis [19,33]. Our results verify these findings that SVV infection induced nearly all of hnRNP A1 to relocalize to the cytoplasm (Figure 2a-2b). Nuclear and cytoplasmic isolation assays further verified the redistribution of hnRNP A1, which was more noticeable in SVV-infected than mock-infected BHK-21 cells. Precisely what interactions cause the translocation of hnRNP A1 remain to be characterized, and it will be interesting to determine which cytoplasmic factors induced by SVV infection are involved in the hnRNP A1 retention in the cytoplasm. Research data revealed that hnRNP A1 binds to the 5ʹ untranslated region (UTR) of EV71 and participates in internal ribosome entry site (IRES)-mediated translation, and, moreover, the redistribution of hnRNP A1 in the EV71-infected cells promoted IRES-dependent translation [24]. Relocalization of hnRNP K in the cytoplasm results in interaction with the viral 5ʹ UTR to regulate EV71 replication [39]. The subcellular redistribution of hnRNP M during poliovirus and coxsackievirus infection was similar to that of hnRNP K and hnRNP A1 in other picornavirus-infected cells [24,38,39]. Viral RNA synthesis is initiated by interaction of hnRNP C with poliovirus polypeptide 3 CD and the 3ʹUTR of negative-strand RNA [40], indicating that this is a common replication strategy of picornaviruses to relocalize RNA-binding proteins [35]. We further found that hnRNP A1 colocalized with viral dsRNA in the cytoplasm (Figure 2c). It is possible that hnRNP A1 interacts with the viral genome to regulate viral replication. However, further studies on this interaction are needed to reveal the precise contributor of hnRNP A1 to SVV replication.

EV71 infection induces hnRNP A1 degradation through proteolytic cleavage by 3 C^pro^ [34]. The mutant type of 3 C^pro^ (C147S) loses proteolytic activity and cannot cleave hnRNP A1 [34]. In the present study, hnRNP A1 was mostly degraded after SVV infection at 9 hpi in BHK-21 cells (Figure 1a-1b). The cleaved band of hnRNP A1 was not visible during SVV infection (Figure 1a, 1d), and it was not cleaved in cotransfected GFP-tagged SVV protein-expressing plasmids and HA-hnRNP A1 cells *in vitro* (Figure 3a). This indicated that the integrity of hnRNP A1 is necessary for SVV infection, the cleaved hnRNP A1 is not an essential factor. Poliovirus 2A protease and 3 C^pro^ cleaves and degrades specific nuclear pore complex components, such as nucleoporin 62, which involves the inhibition of the nuclear import of proteins [35,41–43]. The nucleocytoplasmic transport of nuclear proteins is most likely due to disruption of the nuclear pore complex (NPC) structure, which leads to relocalization and facilitates viral translation [35,42,44]. Therefore, we speculated that regulation of SVV replication may be caused by changing the structure and function of NPC, including alterations in nuclear membrane integrity and permeability, then changed the nucleocytoplasmic transportation of hnRNP A1.

The replication of picornaviruses in the cytoplasm, 3 C, or its precursor form, 3 CD, must enter the nucleus of the infected cells dependent on a 3D nuclear localization signal [45]. SVV 3 C^pro^ exhibited cytoplasmic and nuclear distribution, and hnRNP A1 was mainly localized to the nuclei (Figure 3c-3d). After cotransfection, hnRNP A1 was redistributed and colocalized with GFP-3 C in the cytoplasm (Figure 3d). In GFP-transfected cells, HA-hnRNP A1 did not respond to the GFP vector (Figure 3d). This demonstrates that SVV 3 C^pro^ was responsible for relocalization. SVV 3C^pro^ degrades and cleaves numerous cellular innate immunity factors to promote viral replication; these include targeted nuclear factor-κB (NF-κB), interferon regulatory factor 3/7 (IRF3/7), and retinoic acid inducible gene I (RIG-I) for degradation, cleaved mitochondrial antiviral-signaling protein (MAVS) and TRAF family member-associated NF-κB activator. In addition, the conserved catalytic residues of 3C are involved in degradation and cleavage [6–8,10,34]. The degradation of RIG-I is dependent on the caspase pathway. Research data have shown that poliovirus 3C targetd hnRNP M for degradation and cleavage, whereas a catalytically inactive 3C could not degrade and cleave hnRNP M [38]. In this study, we found that 3C^pro^ degraded hnRNP A1 in a dose-dependent manner (Figure 4a-4b). We constructed three mutants lacking the protease activity in 3C^pro^. Interestingly, the level of hnRNP A1 recovered significantly when the enzyme activity of 3C^pro^ was lost (Figure 4c-4d), indicating that the degradation depended on the conserved catalytic residues of 3C^pro^. The pathway involved in 3C^pro^-mediated degradation was determined in the presence of inhibitors of caspases, proteasomes, and lysosomes, including Z-VAD-FMK, MG-132, and NH_4_Cl, respectively. Abrogation of the proteasome activity significantly restored the integrity of hnRNP A1, while inhibition of caspase activity and lysosome activity could not alleviate the degradation (Figure 4f-4g). This suggests that SVV 3C^pro^ degrades hnRNP A1 depending on its protease activity.

The involvement of hnRNP A1 in SVV replication was determined by siRNA-mediated knockdown and lentivirus-mediated overexpression. After transfection with two different siRNAs targeting hnRNP A1, the expression of hnRNP A1 was significantly decreased (Figure 5a). In siRNA-knockdown cells, the level of VP1 was significantly inhibited, and the virus titer was decreased (Figure 5c-5e). Similarly, transfection with hnRNP M-specific siRNAs significantly impaired poliovirus infection in HeLa cells [38]. In addition, picornaviruses hijack hnRNP M to promote replication [38]. In hnRNP K knockdown cells, the viral yield of EV71 was greatly reduced and viral RNA synthesis was postponed [39]. Moreover, hnRNP K is a cellular factor required for the replication of Dengue virus and Junín virus, as evidenced by siRNA-mediated silencing and overexpression [46]. These results indicate that the hnRNP family is a crucial host factor involved the replication of picornaviruses. On the other hand, we used a lentivirus-delivery system to stably upregulate the expression of hnRNP A1 in BHK-21 cells and found that hnRNP A1 facilitates SVV replication in BHK-21 cells (Figure 6). SVV infection was shown to degrade the hnRNP A1 which benefits its growth, this is due possibly to that 3C^pro^ induces relocalization of hnRNP A1 to the cytoplasm and interacts with viral internal ribosome entry site and/or affects the function of a certain protein or an mRNA-encoded protein thereby contributing to SVV replication. Overall, these findings indicate that hnRNP A1 is a crucial host factor for SVV infection.

In conclusion, the results presented here reveal the translocation and degradation of cellular hnRNP A1 during SVV infection and the involvement of the hnRNP A1 in SVV replication. SVV 3C^pro^ contributes to the relocation and degradation of hnRNP A1 in the cultured cells, which is dependent on its protease activity. Taken together, these findings provide important insights into understanding the mechanism of SVV replication for developing the potential control strategies for SVV infection.

## Data Availability

The data that support the findings of this study are available from the corresponding author upon reasonable request.
